# Mouse Genome Informatics (MGI): reflecting on 25 years

**DOI:** 10.1007/s00335-015-9589-4

**Published:** 2015-08-04

**Authors:** Janan T. Eppig, Joel E. Richardson, James A. Kadin, Martin Ringwald, Judith A. Blake, Carol J. Bult

**Affiliations:** Mouse Genome Informatics, The Jackson Laboratory, Bar Harbor, ME 04609 USA

## Abstract

From its inception in 1989, the mission of the Mouse Genome Informatics (MGI) resource remains to integrate genetic, genomic, and biological data about the laboratory mouse to facilitate the study of human health and disease. This mission is ever more feasible as the revolution in genetics knowledge, the ability to sequence genomes, and the ability to specifically manipulate mammalian genomes are now at our fingertips. Through major paradigm shifts in biological research and computer technologies, MGI has adapted and evolved to become an integral part of the larger global bioinformatics infrastructure and honed its ability to provide authoritative reference datasets used and incorporated by many other established bioinformatics resources. Here, we review some of the major changes in research approaches over that last quarter century, how these changes are reflected in the MGI resource you use today, and what may be around the next corner.

## Introduction

The mouse holds special status as a laboratory research animal and is the predominant species used for studying human hereditary diseases. The combination of its characteristics as a small mammal (making it an economical and easily kept laboratory species), the fact that it historically developed as a genetic tool which translated into an extensive and accurate genetic map, the accessibility of all of its life stages to biological inquiry, its genetic and genomic closeness to human, its approximation to the human in physiology and disease susceptibilities and mutations, and the ability to manipulate its genome through molecular intervention and breeding, have made the laboratory mouse preeminent in studies of human biology and disease.

The Mouse Genome Informatics (MGI) resource evolved from the progression and accumulation of knowledge in the international research community and the rapidly growing data on molecular biology. Its beginning sprang from the reality that no longer could individual researchers reasonably keep current with the entirety of mouse biology and genetics without new information aids. Previous ubiquitous tools for exchanging knowledge about the laboratory mouse (e.g., Mouse News Letter (1949–1990), Strains Characteristics compendiums, annual manually constructed genetic linkage maps, and periodically published books such as *Genetic Variants and Strains of the Laboratory Mouse* (Green 1981; Lyon and Searle 1989; Lyon et al. 1996) were no longer enough to keep a researcher abreast of current and exploding data about the biology and the genome of the mouse.

In this review, we first briefly touch on the early days of mouse biology and genetics and then set the stage at which the MGI project began. We then trace its milestones and development over time, relative to what was happening in biological and genomic sciences and how MGI’s plans and progress were shaped by biological and technological changes. Finally, we describe the current MGI and comment on MGI’s next evolutionary steps.

## Mouse genetics: early landmarks

The mouse has been a commensal species with humans for thousands of years. Paintings of ancient oriental courts show mice kept as pets and mouse fancier organizations that bred and showed mice were thriving by the 1800s (Royer 2015). In the late 1800s–early 1900s Abby Lathrop, a famous mouse breeder and fancier, kept many rodent colonies and sold mice as pets, as well as supplying mice to scientists for research purposes. She also collaborated in research projects using her well-pedigreed mouse stocks. Many of today’s existing laboratory inbred strains can trace their ancestry to Ms. Lathrop’s stocks (Steensma et al. 2010).

The mouse got its foothold experimentally in the early 1900s, soon after Cuénot (1902) showed that Mendelian genetics was applicable to mammals. The first inbred strain, DBA, was developed by CC Little beginning in 1909, working on the hypothesis that cancer was hereditary (Little and Tyzzer 1916). And, at about the same time, William Castle made crosses to study the segregation of coat color in mice (Castle and Little 1910). The first genetic linkage in mice was reported by JBS Haldane et al. (1915). For more on the foundation and history of mouse genetics, which is beyond the scope of this review, see, for example, books by Silver (1995) and Guénet et al. (2015), and review articles by Paigen (2003a, b).

## Motivating MGI: the time and the place

By the late 1940s, the global community of mouse research laboratories was still relatively small, but the character of the community was already established as highly cooperative and collaborative. In this decade, 43 publications on mouse genes and heredity^1^ appeared in print. Mouse News Letter, an informal bi-annual newsletter of short research reports, local laboratory news, lists of known and newly discovered genes, and an annual composite genetic map, came into being in 1949. As technologies changed in the 1970s and 1980s (e.g., the advent of biochemical genetics and molecular biology), the rate of data accumulation greatly accelerated, as did the number of researchers involved in biological research worldwide. In 1990, Mouse News Letter was renamed Mouse Genome and merged with *Mammalian Genom*e in 1997. The journal *Mammalian Genome* (Springer) was initiated in 1991 coincident with the establishment of the International Mammalian Genome Society as the official journal for the new society.

With this transition to mouse as a major research species, the rapid accumulation of genetics/genomics knowledge, the quick assimilation of new biological technologies, and the applications of new disciplines to biological studies, there were many ideas and attempts at better collation, systematic organization, establishment of semantic standards, and use of computers to handle, process, analyze, and archive the rapid data accumulation. These first databases also were faced with rapid dynamic changes in computer capabilities, variable access of biologists to computer resources, and limitations at the individual or institutional level in availability of computer hardware, knowledge, and internet services.

## Early beginnings of MGI: 1989–1992

**Fig. 1:**
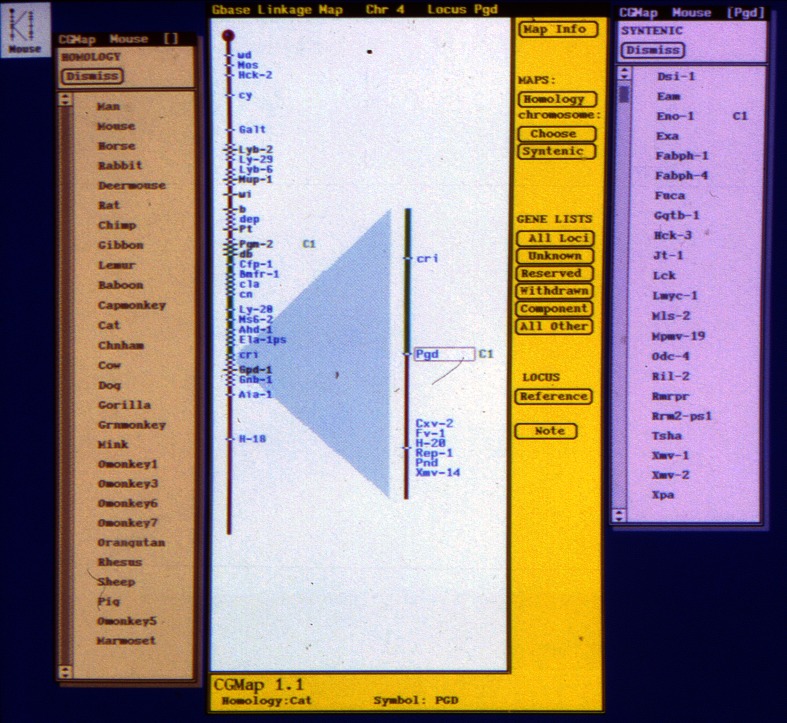
Data visualization using the Encyclopedia of the Mouse Genome software. This view displays data for the *Pgd1* gene on Chromosome 4. The* left panel * shows mammalian species with published homologs. The* center panel *displays Chromosome 4, with the region around *Pgd1* expanded. Note that not all loci are visible in the whole chromosome view due to gene density. The* right panel *displays the syntenic loci for Chromosome 4. Options buttons to view additional panels include gene lists (all or selected subsets) and references

The first incarnation of what would become MGI was initiated in 1989, as a program project grant from the then National Center for Human Genome Research^2^ to JH Nadeau, LE Mobraaten, and JT Eppig entitled “Multilevel Analysis and Display of Mouse Genome Data.” The goal of this project was to use existing specialized databases developed by investigators at The Jackson Laboratory to provide the international mouse community with an interactive tool with visual displays that utilized data from these resources simultaneously and provided a unified view.^3^ The major output of the Multilevel Analysis and Display of Mouse Genome Data project was dubbed the “Encyclopedia of the Mouse Genome” (Fig. 1) and was distributed semi-annually to about 300 investigators worldwide via postal mail on floppy disks.

The initial Encyclopedia of the Mouse Genome displayed chromosome maps, where each tick on the map could be expanded to show more genes (the map was dense even then, relative to computer screen size, with nearly 800 genes localized). In addition, one could visualize cytogenetic maps, human homologs, and access supporting references. The Encyclopedia was developed under SunView and only useful to those with access to an appropriate Sun Workstation. Later, the Encyclopedia software was ported to the OpenLook environment in 1991, a Macintosh version was released in 1993, followed by a platform independent version in 1995, and ultimately the Encyclopedia was available through the early Mouse Genome Database website. The Encyclopedia of the Mouse Genome was a finalist for the Smithsonian Computerworld Award in the Innovation in Information Technology category in 1992 and in 1995 received another Smithsonian nomination in the Medicine category.

## Melding early data components into a unified Mouse Genome Database 1992–1995

The successor “Mouse Genome Informatics” program project brought together the collaborative team of the “Multilevel Analysis and Display” project and the team responsible for developing GBASE (Genomic Database for Mouse Doolittle et al. 1991) led by TH Roderick and MT Davisson. In 1992, the initial goal was to merge the available database resources and build a robust infrastructure to take advantage of the combined data sources on genetic mapping, human–mouse gene homology, molecular reagents and variation (probes, RFLPs, biochemical markers), phenotypic descriptions of known mutants, and references (Richardson et al. 1995).

**Fig. 2:**
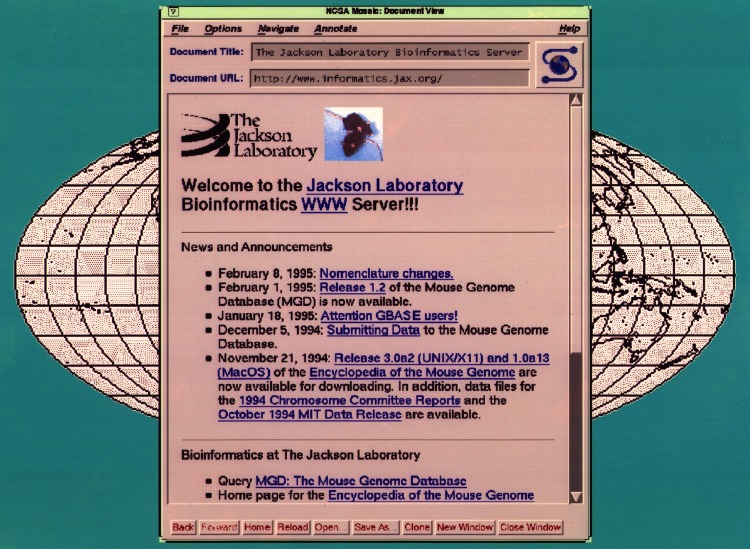
The first worldwide web homepage of the future MGI, 1994. Links to MGD release 1.2 and to the Encyclopedia of the Mouse Genome for Unix and Mac can be seen

Simultaneously, the Worldwide Web gained wider acceptance and the research community rapidly adopted computer technology, which was increasingly desktop-friendly and more intuitive in the programs available for one’s daily work (e.g., easy to use word processing, spreadsheets) and the tools available to analyze clones and sequences. These fortunate co-developments paved the way for developing Worldwide web access to the first Mouse Bioinformatics Homepage and the first online release of the Mouse Genome Database (MGD) in 1994 (Fig. 2). Full integration of the components of the small pre-existing databases would happen over time as the underlying joint schema and common infrastructure was developed.

**Fig. 3:**
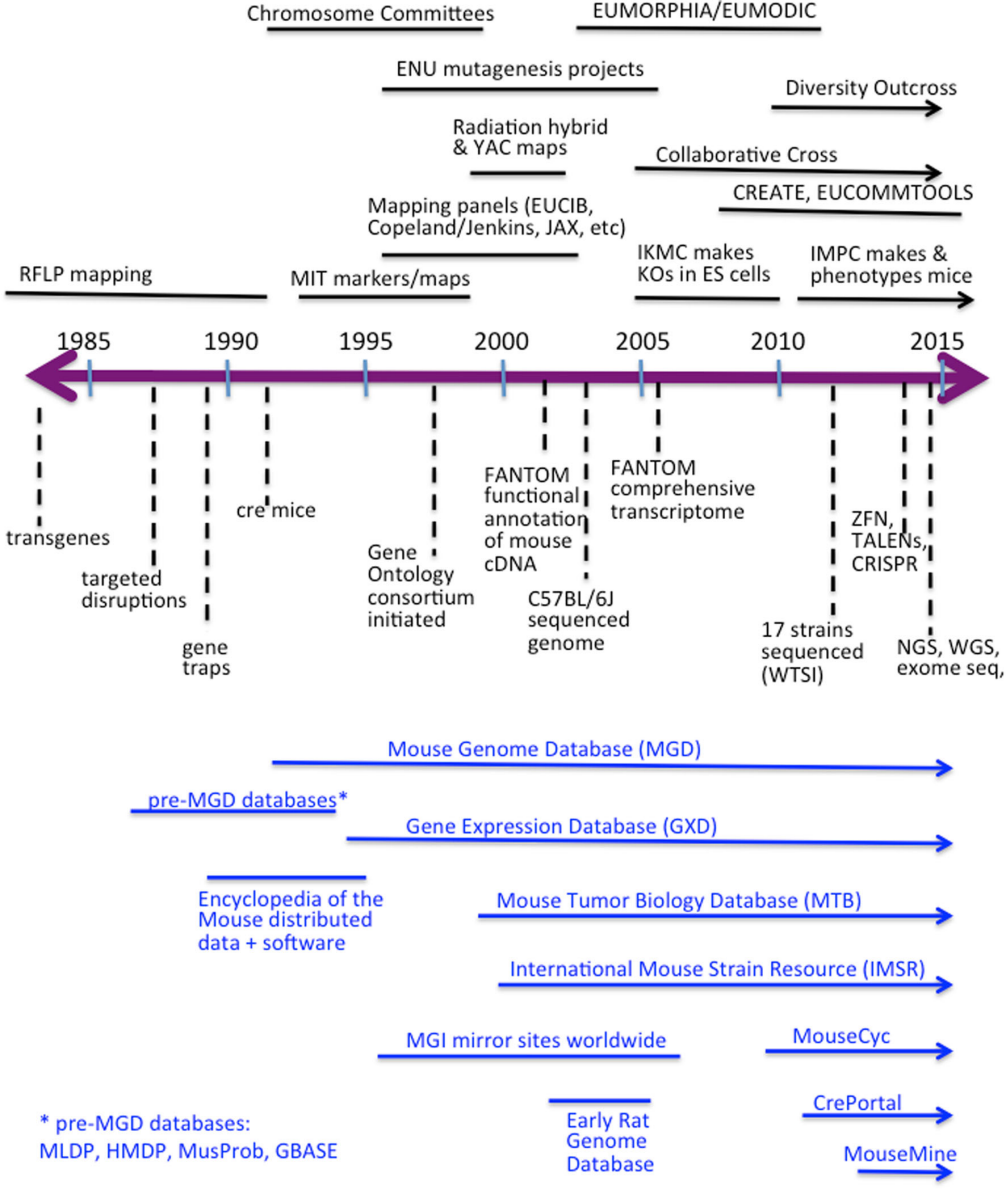
Timeline 1985–2015. Above the timeline are projects and activities in the mouse community. Below the timeline are biotechnology changes and punctuated advances. The bottom section (*blue*) shows how the MGI resource was developed over time. The time ranges are approximate and not drawn strictly to scale

As MGD grew and matured, the scientific community continued to explore new directions that required MGD to continue changing and evolving to accommodate the changing research landscape, a process that continues to this day (Fig. 3). Mouse Chromosome Committees were formed in 1991 to produce collaborative annual reports that included summaries of noted research and consensus genetic maps of the mouse chromosomes, reconciling and combining data from published and unpublished genetic linkage experiments. These Mouse Chromosome Committee reports were published as annual Special Issues of *Mammalian Genome* (“Encyclopedia of the Mouse Genome”) from 1991 to 1998 and the data and consensus maps were made available online through MGD.

## The human genome project years: 1991–2002

The Human Genome Project began in 1991 with a goal to fully sequence the human genome in 10 years. A plan for the first 5 years (1991–1995) is available at http://www.genome.gov/10001477, as are subsequent revisions and reports. At that time, mouse was considered an important model organism and worthy of sequencing. But because its genome was as complicated as human (estimated then to contain 50–100,000 genes), the initial 5-year goal for mouse was limited to developing the genetic map based on DNA markers and to starting to physically map 1–2 chromosomes.

In 1992, Dietrich et al. (1992) reported the first genetic map for mouse using simple sequence length polymorphisms, with 317 markers located along the chromosomes. This quickly expanded to a 7377 marker map by 1996 (Dietrich et al. 1996). A number of large-scale interspecific backcross mapping resources (EUCIB, Rhodes et al. 1998; JAX, Rowe et al. 1994; Copeland/Jenkins, Copeland et al. 1993 and others) peppered the mouse genome with new genetic variants that gave the mouse map a previously unknown level of marker density, allowing virtually all new mutations or sequence fragments to be mapped relative to this new dense map. MGD responded by loading and integrating data from all of these mapping panels and making them accessible and searchable via its web interface.

By 1998, the National Institutes of Health expanded its goals relative to the mouse, and proposed work to lay the basis for finishing the mouse sequence by 2005, with a draft sequence to be available earlier (Collins et al. 1998). As with the human sequencing effort, sequencing the mouse was a high-intensity project, global in reach, and reflected lessons learned from the human effort. The first set of papers describing analysis of the complete mouse sequence for C57BL/6J appeared in 2002 (Mouse Genome Sequencing Consortium 2002).

## Mouse mutagenesis and phenotyping projects

In the years since the human and mouse genome sequences were initially released, there continue to be more and better quality sequence added, periodic re-assemblies of the genomes, and continuous updates to annotations, improving the reliability of these reference genomes. The next questions that clearly could benefit from large-scale organized studies were to discover the functions of the genes, individually and collectively, and how they are related to hereditary diseases and susceptibilities.

### Forward genetics: ENU mutagenesis

Between 1997 and 2005, many large-scale programs began worldwide to mutagenize and create new defined mutations in mice, largely using ENU (*N*-ethyl-*N*-nitrosourea) for the mutagen and following various breeding schemes to uncover new phenotypes and identify gene mutations. Mutants could be systematically screened for phenotype (c.f. Gondo et al. 2010; Justice et al. 1999; Kile and Hilton 2005; Goldowitz et al. 2004). The sticking point was the mapping and identification of the genes mutagenized, since ENU is a random and not targeted mutagen, and exome or whole genome sequencing was not yet economically viable. These programs produced several thousand new mutant alleles in mice that were phenotypically characterized and many localized through traditional linkage mapping methods. Even though most of these large systematic programs are no longer operational, ENU mutagenesis continues, for focused screens such as immunity (Arnold et al. 2012; Caignard et al. 2014), ciliopathy (Damerla et al. 2014), and epigenetics (Daxinger et al. 2013), but now with the advantage of using next generation sequencing technologies to rapidly identify the mutations generated. In addition, current ENU mutagenesis efforts such as those of the Australian Phenomics Facility (Bull et al. 2013), Mutagenetix (Andrews et al. 2012), the Cardiovascular Disease Consortium (Li et al. 2015), and the RIKEN (Sakuraba et al. 2005) mutagenesis effort now routinely sequence G0 progeny and freeze sperm, so that “incidental” mutations (those not of interest to the current program) might be recovered by others seeking novel mutations in their gene(s) of interest.

### Reverse genetics: systematic targeted mutagenesis

From 2005 to 2010, the International Knockout Mouse Consortium (IKMC, Bradley et al. 2012), consisting of KOMP (Knockout Mouse Project, USA), EUCOMM (European Conditional Mouse Mutagenesis Program, Europe), EUCOMMTOOLS (Tools for Functional Annotation of the Mouse Genome, Europe), NorCOMM (North American Conditional Mouse Mutagenesis Project, Canada), and TIGM (Texas A&M Institute for Genomic Medicine, USA) (International Mouse Knockout Consortium 2007; Collins et al. 2007), worked toward a goal of mutating all protein-coding genes in mouse using gene trapping and gene targeting in C57BL/6N mouse embryonic stem (ES) cells. Unlike the forward genetics strategy, known mutations were created with defined molecular constructs, but with completely unknown phenotypes.

In 2011, the International Mouse Phenotyping Consortium (IMPC, Brown and Moore 2012) began generating mice from these ES cell lines and putting them through a broad-based systematic phenotyping pipeline to discover the mutant targeted gene’s phenotypic effects. With several hundred lines successfully analyzed to date, there are, as would be expected, a wide range of interesting phenotypes uncovered (Adissu et al. 2014; White et al. 2013; Bassett et al. 2012). Further detailed phenotypic analyses will be done by individual investigators selecting these mice for study, based on these initial broad-based screens.^4^

MGD now integrates the mutations generated via ENU, the IKMC knockout programs, and the emerging CRISPR/Cas editing technologies, along with their annotated phenotypes to ensure the complete mutagenic picture of the mouse genome. It remains important to characterize allelic series, understanding the effects of null mutations, as well as other variation types (point mutations, in-dels, etc.) in interpreting the many aspects of gene functions and interactions.

## Today’s MGD: from sequence to function, phenotype, and disease models

At its core, MGD provides a set of reference data used widely by researchers and computational biologists. The datasets for which MGD is considered the “gold-standard” and official data source are given in Table 1. The wide use of these MGD high-quality datasets within the greater bioinformatics and bioresources communities emphasizes MGD’s role in representing mouse data and the mouse community in the wider ecosystem of biological informational resources.

**Table 1:** Data for which MGD serves as the authoritative source

Data type	Maintained as
Unified mouse gene and genome feature catalog	Integrated gene predictions from Ensembl, NCBI, and Havana/Vega with MGI curated genes, creating a catalog of features with genome location, unique identifiers, cross-links to other provider identifiers and sequences
Gene Ontology (GO) annotations for mouse	Associations between mouse genes and GO terms
Mouse Phenotype annotations	Associations between mouse genotypes and MP terms
Mouse models of human diseases	Associations between mouse genotypes and human disease terms
Gene-to-nucleotide sequence association	Mapping of genes to their sequences
Gene-to-protein sequence association	Mapping genes to protein products
Mammalian Phenotype (MP) Ontology	Ontology of defined phenotype terms and relationships
Symbols and names for genes and genome features	Nomenclature associated with unified genome feature catalog, nomenclature history and synonyms
Symbols and names for mutant alleles & genome rearrangements	Complete catalog of mutations, with unique identifiers, description of mutant construction and inheritance
Strain designations	Catalog of strains
Sequence Ontology (SO) annotations for mouse	Associations between mouse genome features and SO terms

Careful integration of data from many disparate sources is critical to producing and maintaining these high-quality data. This is accomplished by applying quality control measures to all incoming data, whether originating from the scientific literature, direct data submissions from laboratories, or downloads or files from large-scale projects and other resources. Semantic standards, including vocabularies and ontologies, unify metadata and terminologies among data sources and foster creation of the common annotation sets that are required for robust searching and complete results returns among those diverse data. These integrated and curated data relationships enable discovery of new data relationships and promote hypothesis building.

Broadly, MGD integrates genetic, genomic, variant, functional, phenotypic, and human disease model data essential to biomedical research and makes these data available through a variety of web-based and programmatic interfaces. The core data MGD targets for integration are described elsewhere in this issue of Mammalian Genome and include the canonical catalog of mouse genome features (Zhu et al. 2015); mouse functional annotations (Drabkin et al. 2015); gene orthology for comparative genomics (Dolan et al. 2015); and the comprehensive catalog of mouse mutant alleles and their phenotype and disease model associations (Bello et al. 2015). We also recommend the reader consult a recent review of MGD in Genesis (Eppig et al. 2015a) and the annual update in Nucleic Acids Research (Eppig et al. 2015b).

**Fig. 4:**
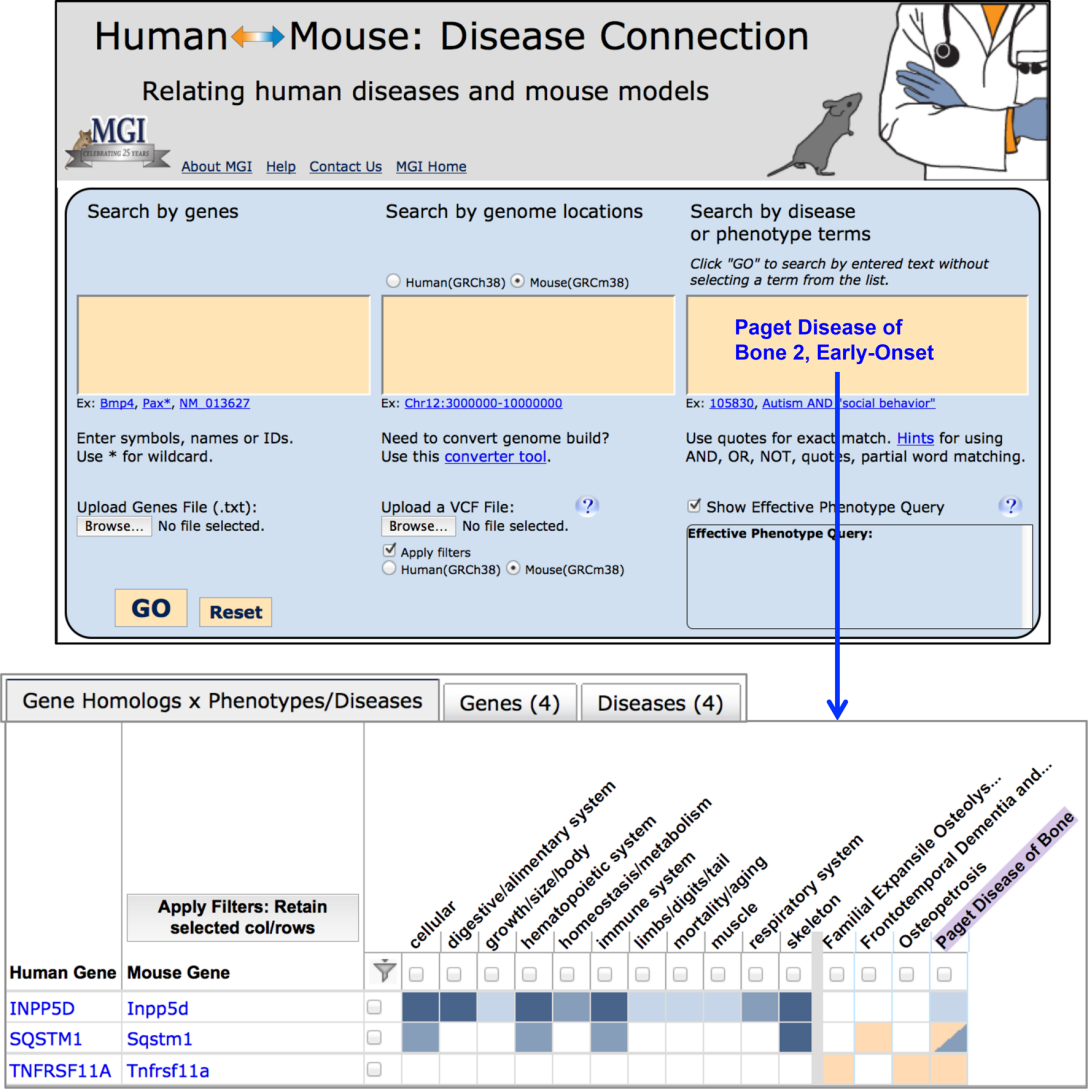
Human–Mouse Disease Connection (HMDC), www.diseasemodel.org. The* top panel * shows the upper portion of the HMDC homepage with 3 distinct search boxes to allow searching by mouse or human genes, genome locations, or disease or phenotype terms. Note that options are provided to upload a gene file or a VCF file to use as search parameters as well. In this example, Paget Disease of Bone 2, Early-Onset was entered in the disease/phenotype term box. The* lower panel * shows the resulting grid display where human and mouse orthologs are shown in rows and phenotypes and diseases are shown in columns.* Blue* indicates mouse data;* orange * indicates human data. The highlighted Paget Disease of Bone column shows both human *SQSTM1* and mouse *Sqstm1*, respectively, are associated to the disease. Mouse gene *Inpp5d* and human gene *TNFRSF11A* are associated to this human disease as well, but not coincidentally. These data suggest that mice with mutations in *Tnfrsf11a* should be examined for phenotypes correlated to human Paget Disease of Bone 2 and that human patients with Paget Disease of Bone 2 phenotypes might be checked for mutations in the *INPP5D* gene

In addition, in MGD’s efforts to better serve the clinical translational, and comparative research communities, the recently released Human–Mouse Disease Connection portal is being refined and expanded. This interface is designed to take full advantage of MGD’s integrated data on mouse mutant phenotypes and the MGD curated set of mouse models of human disease. These data, coupled with human–mouse orthology data and human gene-human disease association data are used to provide a visualization tool that summarizes known relationships and highlights potential new disease candidate genes for human and potential new mouse genes that might be engineered as future disease models (Fig. 4).

## More than MGD: MGI as an integrated system

As MGD grew and developed, there was impetus to integrate new biological areas that complemented the MGD project scope. These spawned additional data resource projects and the development of the MGI resource as an umbrella integrating several additional programs.

### Gene expression database for mouse development (GXD)

The gene expression database for mouse development (GXD) began in 1994, initiated as a pilot project with funding from the Keck Foundation. The early prototype became a Eunice Kennedy Shriver National Institute of Child Health and Human Development funded database program from 1995 onward under the leadership of Ringwald et al. (1997). GXD first appeared on the MGI website in 1996 as a stand-alone entity, and became fully integrated with MGI in 1998. This important step gave users access to simultaneous searching of MGD content (gene function, phenotypes, etc.) along with temporal-spatial expression specific data. For a description of current GXD implementation in MGI see Smith et al. (2015).

### Gene ontology (GO)

In 1998, MGI, along with the Saccharomyces Genome Database (SGD) and the Drosophilia Genome Database (Flybase), were independently wrestling with ways to represent gene function within our respective data resources. The consensus of a number of meetings and debates about the underlying biology and how to organize a unified species-independent effort led to the formation of the Gene Ontology (GO) (Gene Ontology Consortium 2000). Annotation of function to mouse genes and gene products using GO has been an integral part of the MGI resource since its inception. For a description of GO implementation in MGI see Drabkin et al. (2015).

### Cre (Recombinase) portal

Conditional mutagenesis allows for the spatial and temporal control of genetically engineered modifications using site-specific recombinases, of which cre is currently the most widely used. The MGI Cre Portal provides specificity data for cre expression and links to reported phenotypes using specific cre constructs to aid in selecting the best cre transgene or knock-ins for one’s experiments. First brought online in 2011 (Blake et al. 2011; Murray et al. 2012), MGI’s Cre Portal provides searching and downloading capabilities, and links to IMSR for locating cre resources in public repositories.

### International mouse strain resource (IMSR)

The International Mouse Strain Resource is a catalog of available mouse resources worldwide. First made available in 1999, this catalog is continuously updated by participating repositories that regularly contribute full listings of their holdings, including live mice, cryopreserved embryos and gametes, and mutant ES cell lines. Users can search IMSR directly and, in addition, MGI mutant allele pages link to corresponding IMSR holdings for the phenotypes being viewed (Eppig et al. 2005). Each strain listed provides direct links to repositories for ordering mouse resources. For a description of current IMSR implementation see Eppig et al. (2015c).

### MouseMine

MouseMine, first released in 2013, is an instance of InterMine (Smith et al. 2012) that provides a new access method to MGI data. MouseMine provides flexible querying, pre-defined templates, and iterative refinements of results. While not as intuitive as the MGI web interface, it is much more powerful for developing customized datasets and addressing queries not possible through the MGI web. Data enrichment analyses are also included. For a description of MouseMine see Motenko et al. (2015).

### Mouse tumor biology database (MTB)

The Mouse Tumor Biology Database appeared online in 1998 (Bult et al. 1999). MTB’s goal is to facilitate the selection of strains for cancer research and provide a platform for mining data on tumor development and patterns of metastases. Initial data emphasis for MTB centered on genetically engineered mouse models of cancer and documenting the influence of genetic background on cancer phenotypes. Recent changes in direction include expanding data to large-scale analysis [e.g., from IMPC and the (Collaborative Cross Consortium 2012) and Diversity Outcross panels (Churchill et al. 2012) and incorporation of patient derived xenograft data]. For a description of MTB see Bult et al. (2015).

### MouseCyc

MouseCyc is a database of curated biochemical pathways for mouse (Evsikov et al. 2009) based on the Pathways/Genome Database tool (Karp et al. 2010). MouseCyc allows users to browse and search the pathway data and create a metabolic map.

## MGI today and beyond

### MGI’s 25th birthday

**Fig. 5:**
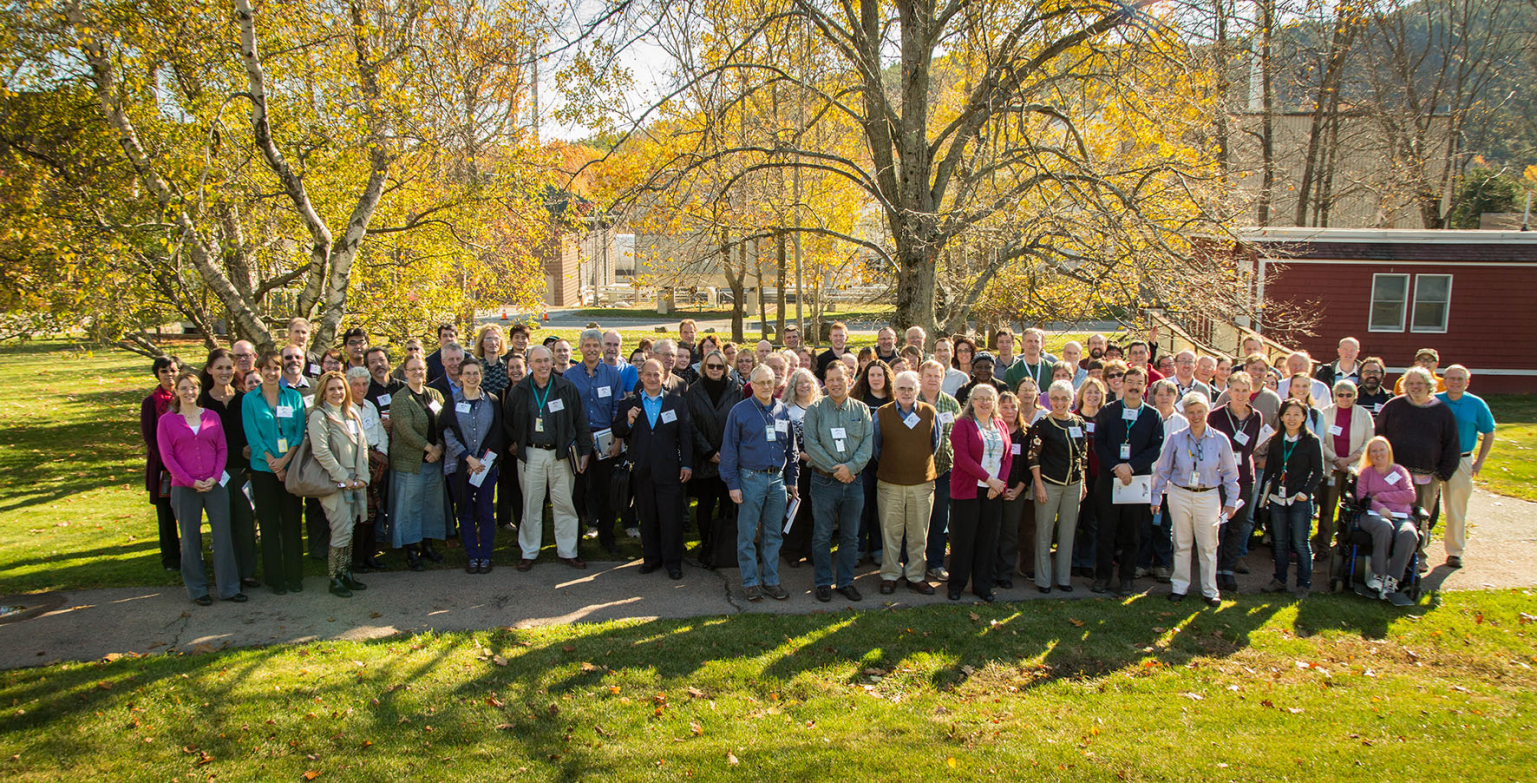
The MGI 25th celebration. Photo of participants and attendees at the celebration of MGI’s 25th year, October 30, 2014, Bar Harbor, Maine

On October 30, 2014, MGI held a 25th birthday celebration at the Jackson Laboratory (Fig. 5). This event highlighted both where MGI started, as well as its journey to the present. Several clear themes emerged from the invited seminar presentations, discussions of participants, and the view of the “big picture” over the 25 years of this program. These included that MGI

**Fig. 6:**
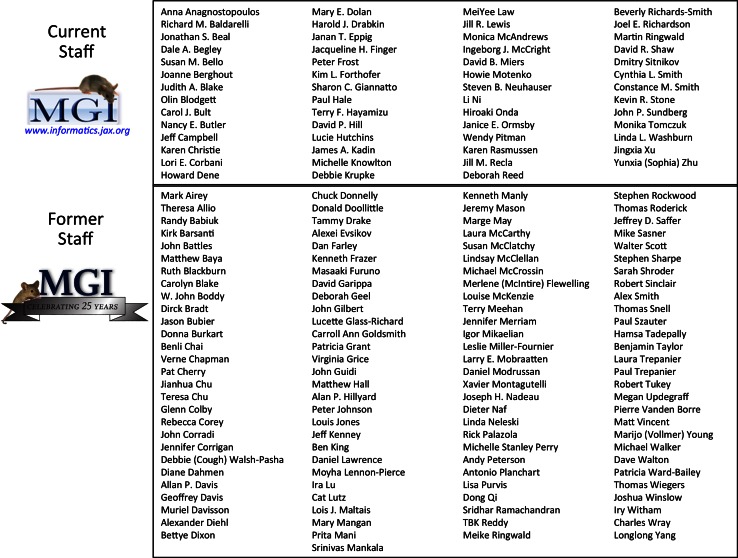
Staff of MGI over its first 25 years. The 168 members of the MGI team, 1989–2015

- has undergone remarkable changes over its history;
- evolved and adapted to dramatic changes in biological techniques, computer technology, and community expectations;
- successfully responded and delivered data, access, and analysis needs for supporting mouse research and mouse models research;
- plays a key role in the global bioinformatics infrastructure, providing authoritative source for many mouse sets data incorporated into other resources and used as a basis for computational work; and
- is increasingly central to translational discovery through its work to integrate unique data resources and represent relationships between mouse and human genes, mouse phenotypes and specific genotypes and strains, human diseases and causative human gene mutations, and mouse models and human disease.

In the future, MGI envisions more change and adaptation. With the caveat that progress in biological discovery and biotechnology is a moving target, some challenges foreseen include

- more translational and computational resources and applications of mouse data;
- expansion of human–mouse phenotype comparisons to aid new disease model development;
- integration of Collaborative Cross and Diversity Outcross population data for dissecting complex phenotypes and multigenic traits;
- enhanced representation of non-coding RNAs and other emerging genome elements;
- development and extension of data visualizations for ontology relationships, genome comparisons, and interactions among genome features; and
- support for functional genome discovery through enhanced integration of spatiotemporal expression data and species and strain comparative phenotype, sequence, and variant data.

In sum, MGI has flourished in its first 25 years and looks forward to exciting and challenging times ahead as it continues to transform its essence to meet research progress in its next quarter century.

## Footnotes

1 Search of PubMed for 1940–1949 publication dates with terms “mice AND (genetics OR heredity)”.

2 Now the National Human Genome Research Institute.

3 Recall, at that time, the Worldwide Web was not widely accessible and its speed and capacity limited; and most biologists were not yet using computers as a daily and necessary tool. What systems were in use were largely monitors tied to a central server with command line interfaces. Desktop computers were both new and had very limited capabilities gated by their floppy disks, which loaded both programs and data into memory.

4 Note that for IMPC, mice recovered from ES cell lines are first crossed to Cre lines to excise the critical exon in the case of the conditional ready mutations; or to excise the neo insert in the case of deletion mutations. Some portion of the future IMPC phenotyped mice will carry knockout alleles based on CRISPR/Cas editing, rather than the IKMC mutant ES cell lines.
